# Family satisfaction with patient care in ICUs of nonacademic Brazilian public hospitals

**DOI:** 10.1186/cc12483

**Published:** 2013-03-19

**Authors:** J Othero, AB Cavalcanti, JC Mouro, K Normilio-da-Silva, R Pusch, F Moreira, AA Kodama, O Berwanger

**Affiliations:** 1 Hospital do Coração - HCor, São Paulo, Brazil

## Introduction

We are conducting a cluster randomized trial with two parallel arms to evaluate strategies to improve family satisfaction with the care that themselves and their critically ill relatives receive in the ICUs of nonacademic Brazilian public hospitals. Here we report the results of the baseline phase of this trial.

## Methods

In this baseline phase, we interviewed the family member most closely involved with the care of critically ill patients who stayed in the ICU for at least 72 hours. We applied a form with 24 questions divided into four domains: overall ICU experience, communication, decision-making, and questions related to end-of-life care for patients who died in the ICU. Each question scored from 0 (very poor) to 100 (excellent). The form was adapted from the Family Satisfaction with Care in the ICU (FS-ICU 24). As many questions assessed the quality of intensivist care or communication, the interview was applied by a psychologist or a nurse.

## Results

Families of 564 patients were interviewed. A total 45/564 (8.3%) died in the ICU. Most respondents were satisfied with overall ICU experience (mean ± SD score 85.5 ± 11.9). However, family satisfaction with communication (67.8 ± 18.0) and decision-making (69.5 ± 21.1) resulted in somewhat lower scores. Most families of patients who died in the ICU (38/45 (82.6%)) considered that their relative's life was neither extended nor shortened unnecessarily. Also, most of the families believed that their relative did not suffer or suffered little in the ICU (37/46 (80.4%)) and felt supported by the healthcare team (40/46 (87.0%)).

## Conclusion

Most families were satisfied with the care themselves and their critically ill relatives received in the ICU. Also, most relatives of patients who died in the ICU felt that end-of-life care was adequate. Although we believe there is much room for improvement in communication, decision-making and support critically ill patients and their families, as their baseline satisfaction with patient care is quite high, it may be hard to demonstrate substantial improvement after interventions.

